# Supplementary material for: DNA damage independent inhibition of NF-κB transcription by anthracyclines
Source: eLife. 2022 Dec 7;11:e77443. doi: 10.7554/eLife.77443 (PMC9771368; doi:10.7554/eLife.77443)
Supplement: Figure 2—source data 1. — DAVID functional analysis of the RNA-seq data in macrophages stimulated with lipopolysaccharide (LPS) for 4 hr showing representative clusters of genes downregulated by Epirubicin (Epi) and Aclarubicin (Acla). [file elife-77443-fig2-data1.docx]

Figure 2 - table supplement 1.

| DAVID functional analysis | | | | | |
| --- | --- | --- | --- | --- | --- |
|  | **Category** | | | **Term** | **Cluster Enrichment Score** |
| Epi | UniProt Keywords | | Inflammatory response | | 7.13 |
|  | UniProt Keywords | | Immunity | |  |
|  | UniProt Keywords | | Innate immunity | |  |
|  | GO Biological Process | | Immune system process | |  |
|  | GO Biological Process | | Innate immune response | |  |
|  | KEGG PATHWAY | | Cytokine-cytokine receptor interaction | | 6.57 |
|  | GO Biological Process | | Immune response | |  |
|  | UniProt Keywords | | Cytokine | |  |
|  | GO Biological Process | | Cytokine activity | |  |
|  | INTERPRO | | Tumour necrosis factor | | 1.94 |
|  | SMART | | TNF | |  |
|  | INTERPRO | | Tumour necrosis factor, conserved site | |  |
|  | INTERPRO | | Tumour necrosis factor alpha/beta/c | |  |
|  | Go Molecular Function | | Tumor necrosis factor receptor binding | |  |
|  | INTERPRO | | Tumour necrosis factor-like domain | |  |
| Acla | UniProt Keywords | | Immunity | | 26.37 |
|  | GO Biological Process | | Immune system process | |  |
|  | UniProt Keywords | | Innate immunity | |  |
|  | GO Biological Process | | Innate immune response | |  |
|  | GO Biological Process | | Immune response | | 9.85 |
|  | KEGG PATHWAY | | Cytokine-cytokine receptor interaction | |  |
|  | UniProt Keywords | | Cytokine | |  |
|  | Go Molecular Function | | Cytokine activity | |  |
|  | GO Biological Process |  | Cellular response to interferon-gamma | | 3.83 |
|  | GO Biological Process | | Cell chemotaxis | |  |
|  | GO Biological Process | | Chemotaxis | |  |
|  | UniProt Keywords |  | Chemotaxis | |  |
|  | GO Biological Process |  | Chemokine-mediated signaling pathway | |  |
|  | INTERPRO | | CC chemokine, conserved site | |  |
|  | INTERPRO | | Chemokine interleukin-8-like domain | |  |
|  | GO Biological Process | | Neutrophil chemotaxis | |  |
|  | Go Molecular Function | | Chemokine activity | |  |
|  | GO Biological Process | | Lymphocyte chemotaxis | |  |
|  | GO Biological Process | | Monocyte chemotaxis | |  |
|  | GO Biological Process | | Positive regulation of natural killer cell chemotaxis | |  |
|  | Go Molecular Function | | CCR chemokine receptor binding | |  |
|  | GO Biological Process | | Macrophage chemotaxis | |  |
|  | GO Biological Process | | Eosinophil chemotaxis | |  |
