# Supplementary figures and images for: DNA damage independent inhibition of NF-κB transcription by anthracyclines
Source: eLife. 2022 Dec 7;11:e77443. doi: 10.7554/eLife.77443 (PMC9771368; doi:10.7554/eLife.77443)

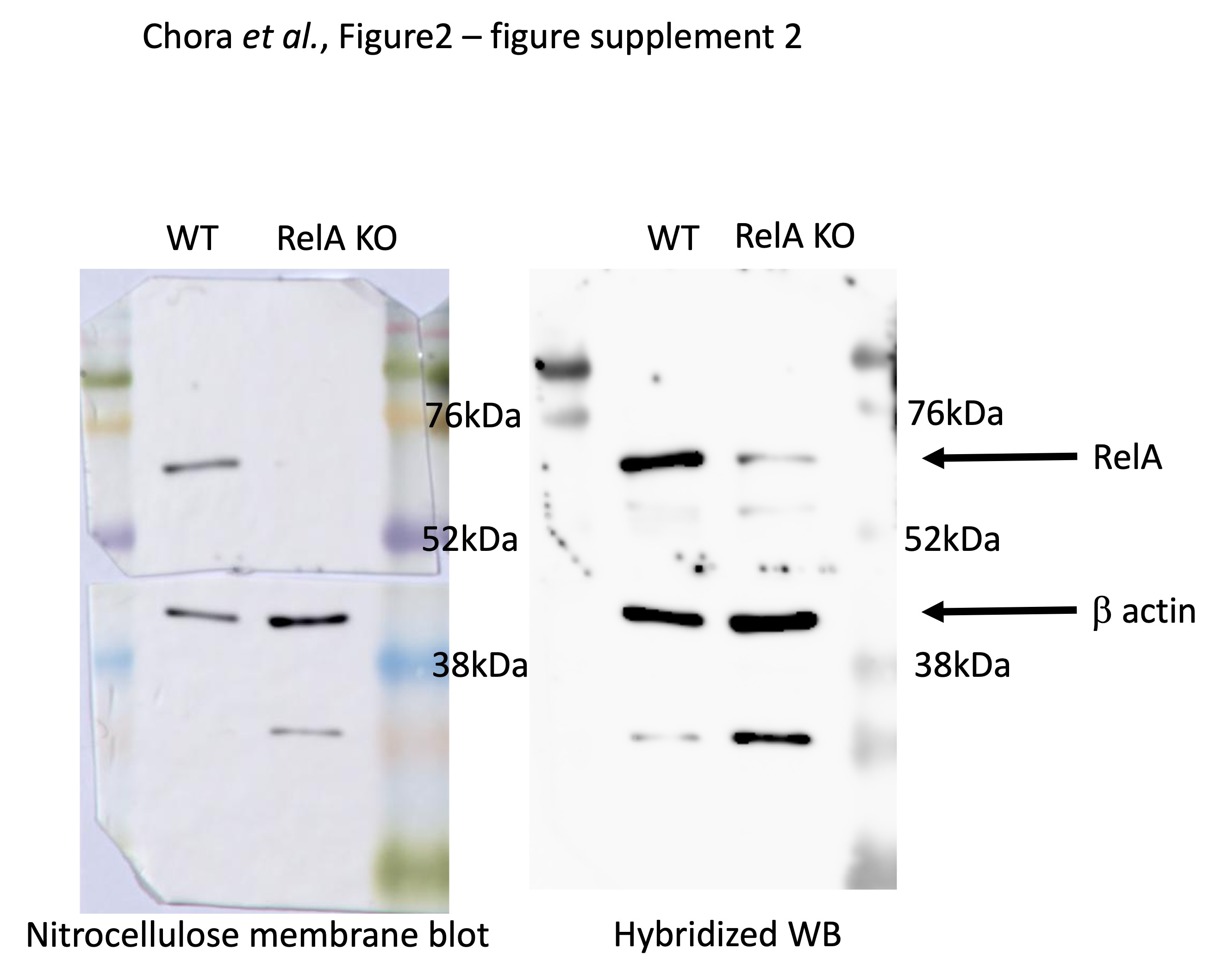

Supplement: Figure 2—figure supplement 2—source data 1. [file elife-77443-fig2-figsupp2-data1.zip › Figure2-figure supplement2 - source data1.jpg]

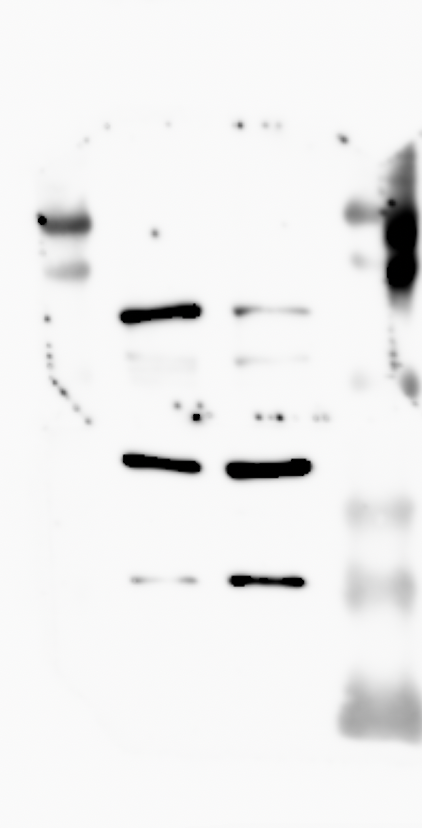

Supplement: Figure 2—figure supplement 2—source data 2. [file elife-77443-fig2-figsupp2-data2.zip › Figure2-figure supplement2 - source data2.png]

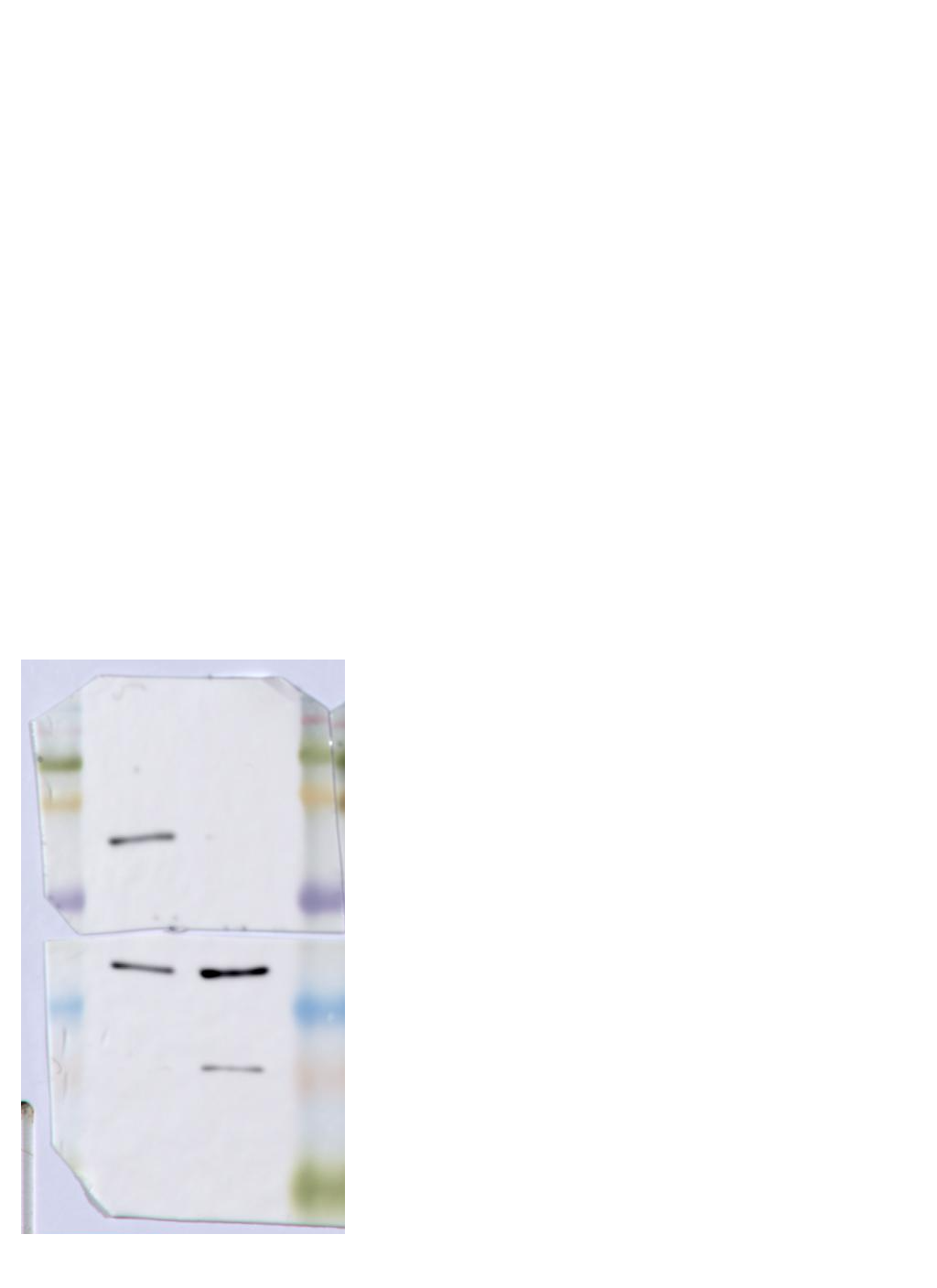

Supplement: Figure 2—figure supplement 2—source data 3. [file elife-77443-fig2-figsupp2-data3.zip › Figure2-figure supplement2 - source data3.png]

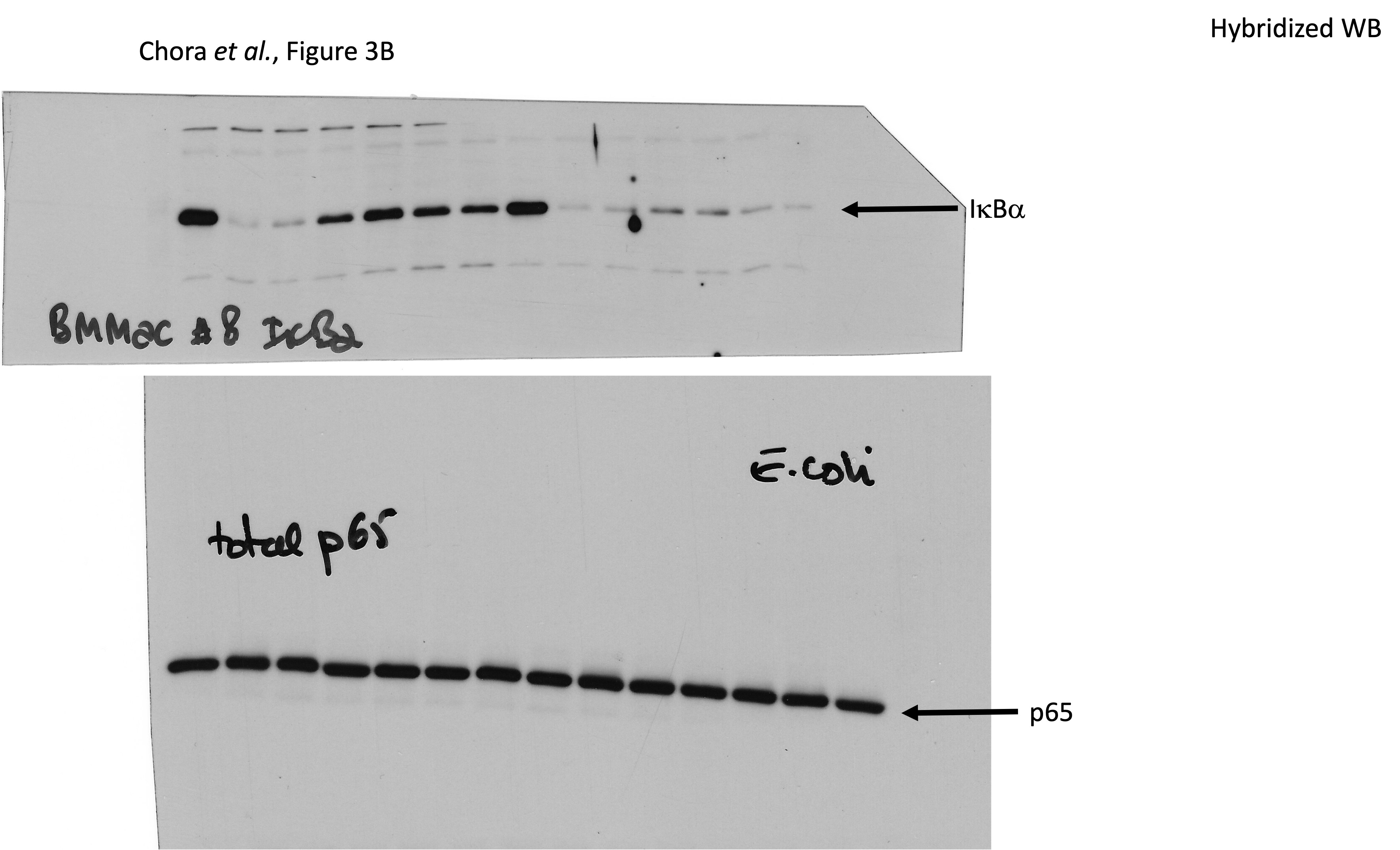

Supplement: Figure 3—source data 1. [file elife-77443-fig3-data1.zip › Figure3 - source data1.jpg]

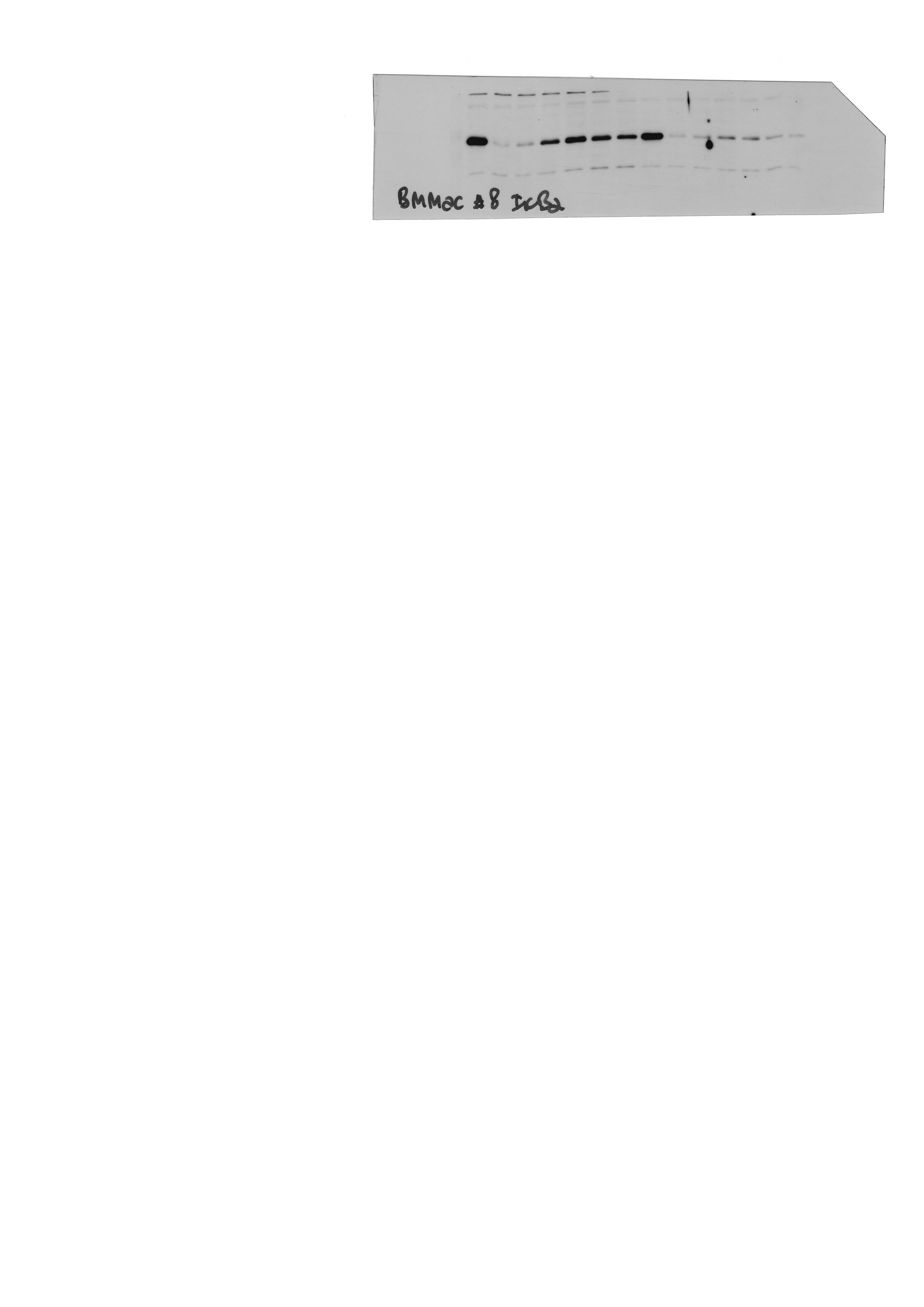

Supplement: Figure 3—source data 2. [file elife-77443-fig3-data2.zip › Figure3 - source data2.png]

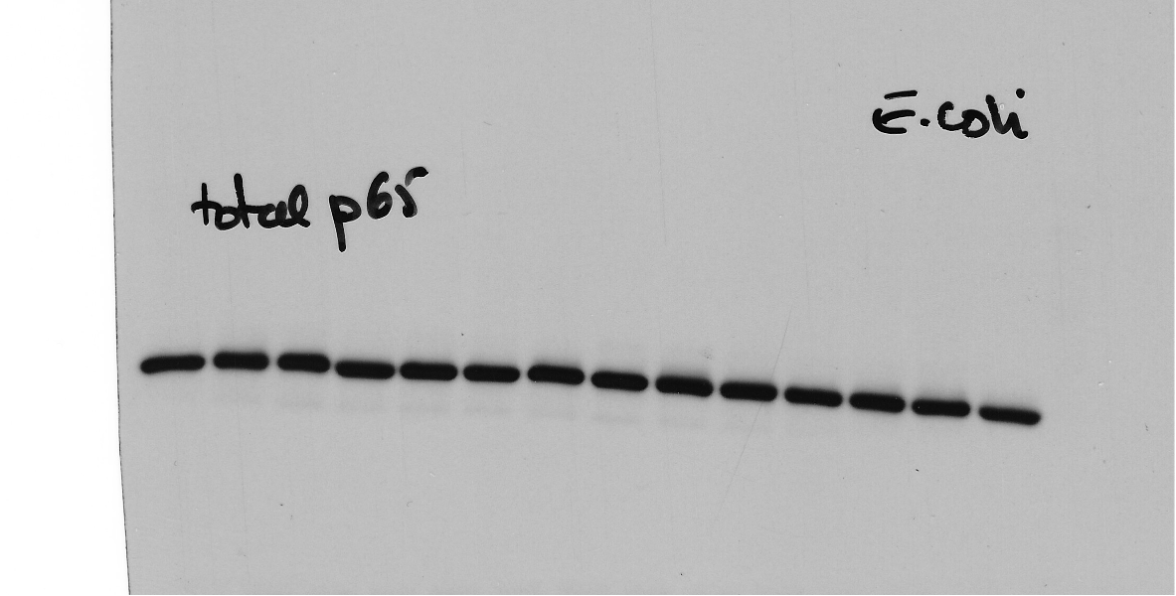

Supplement: Figure 3—source data 3. [file elife-77443-fig3-data3.zip › Figure3 - source data3.png]

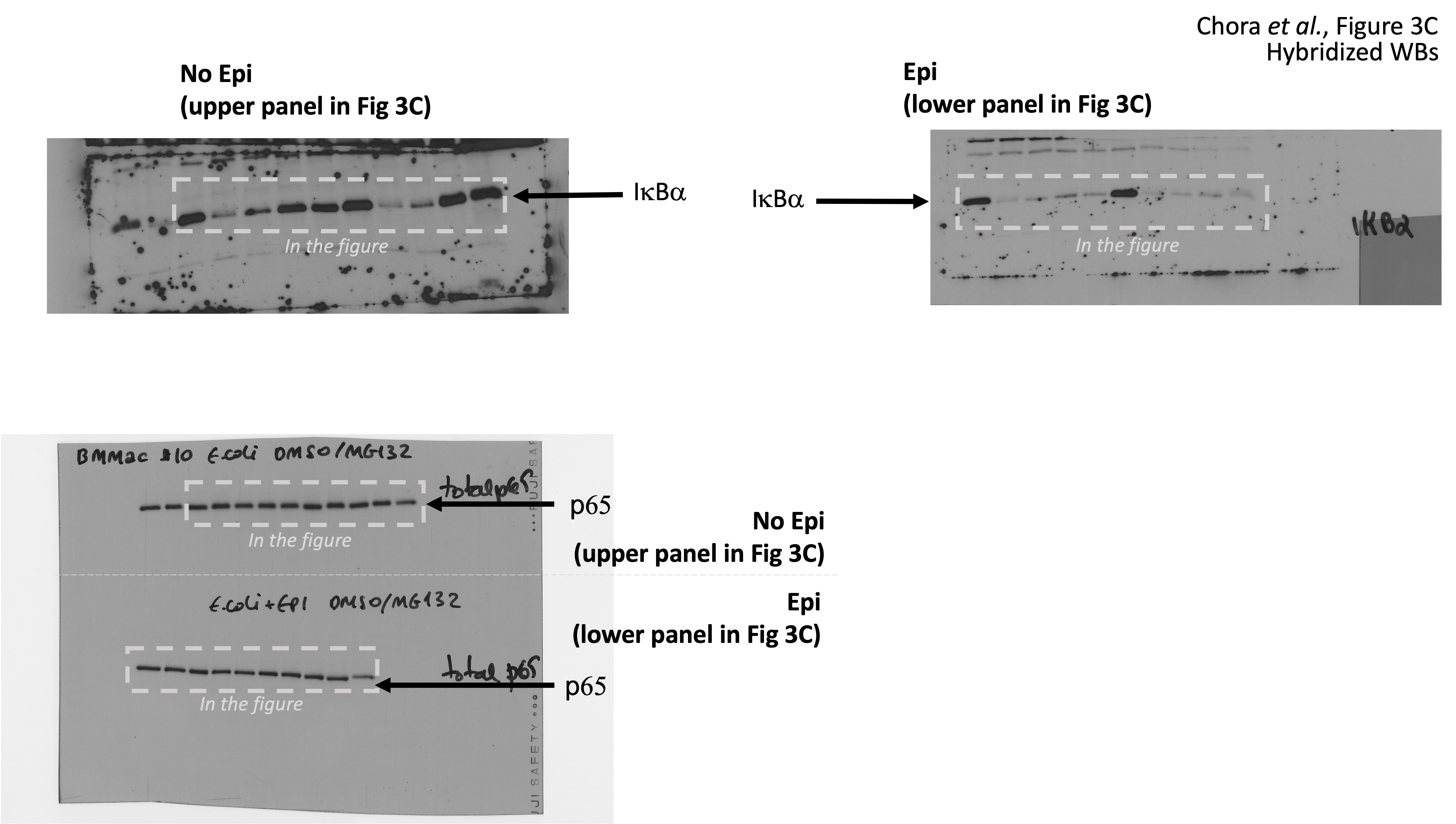

Supplement: Figure 3—source data 4. [file elife-77443-fig3-data4.zip › Figure3 - source data4.jpg]

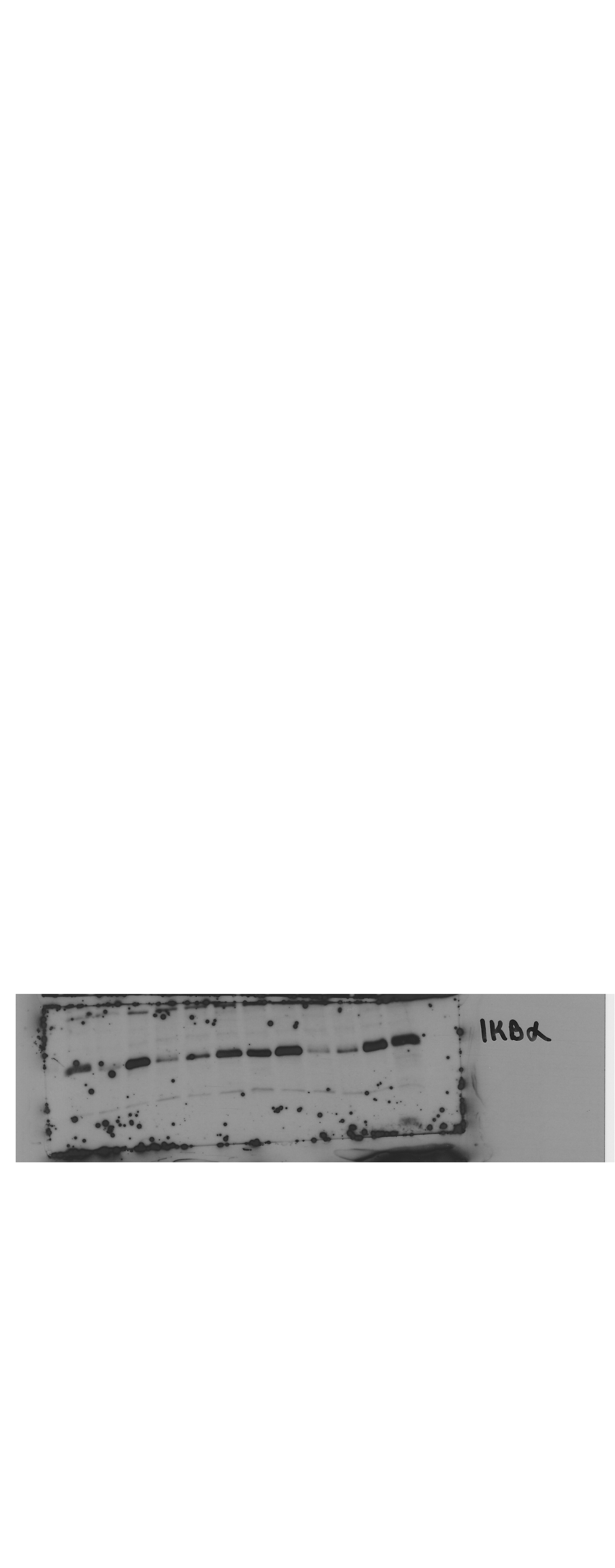

Supplement: Figure 3—source data 5. [file elife-77443-fig3-data5.zip › Figure3 - source data5.png]

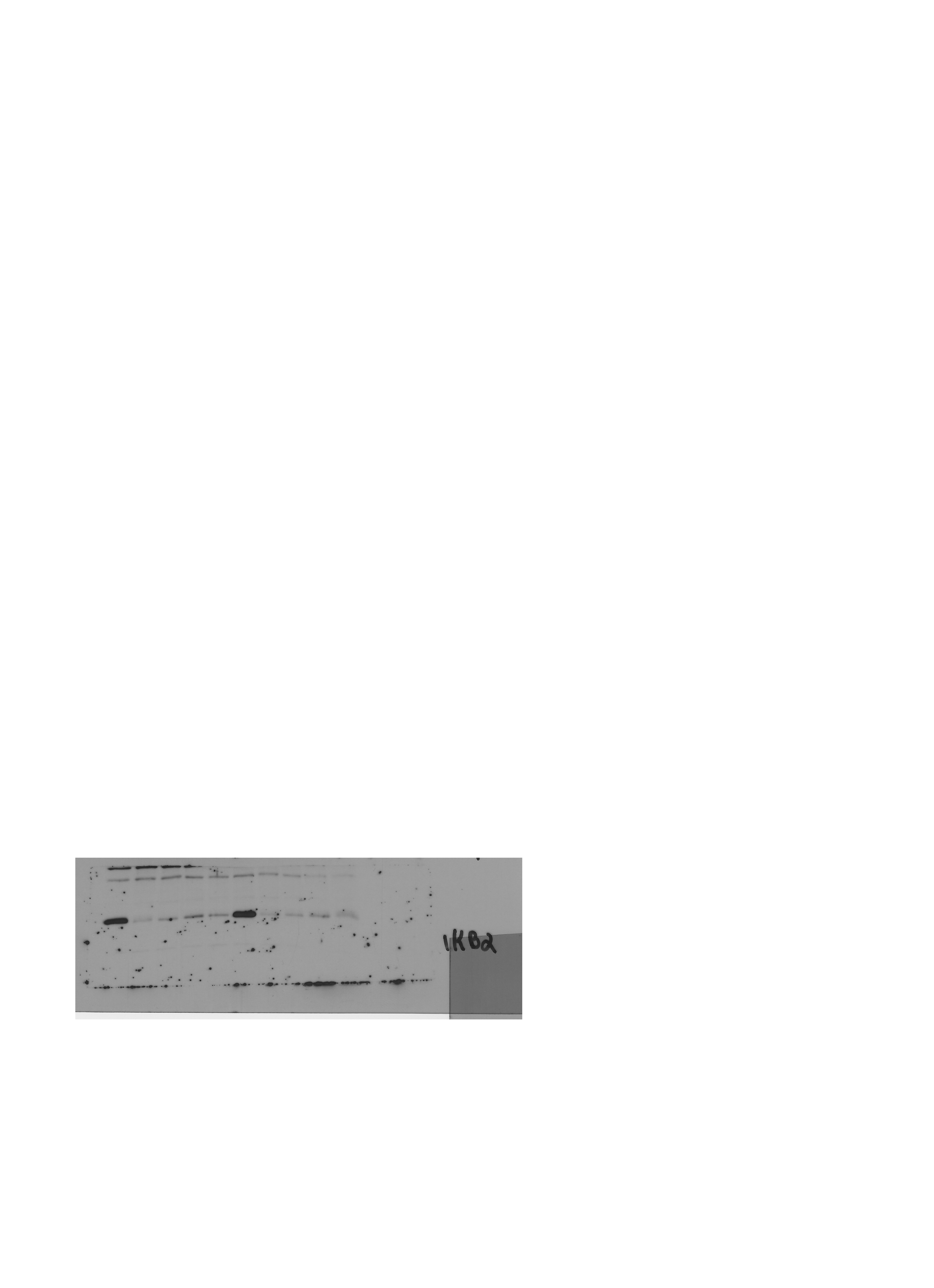

Supplement: Figure 3—source data 6. [file elife-77443-fig3-data6.zip › Figure3 - source data6.png]

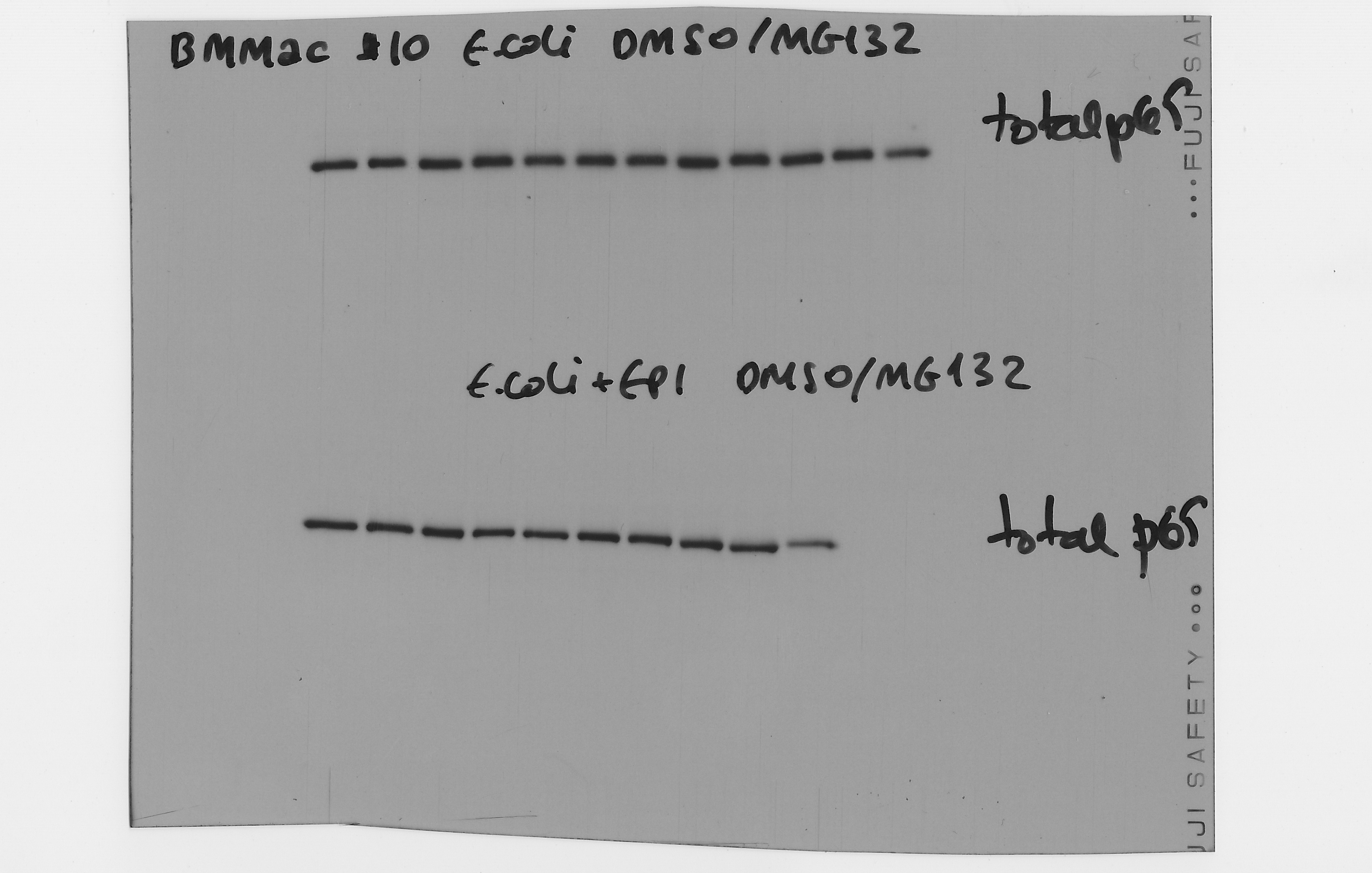

Supplement: Figure 3—source data 7. [file elife-77443-fig3-data7.zip › Figure3 - source data7.png]
