# Supplementary material for: DNA damage independent inhibition of NF-κB transcription by anthracyclines
Source: eLife. 2022 Dec 7;11:e77443. doi: 10.7554/eLife.77443 (PMC9771368; doi:10.7554/eLife.77443)
Supplement: Figure 4—source data 1. [file elife-77443-fig4-data1.docx]

Figure 4 - table supplement 1

| Experiment | MW_exp_ RelA (kDa) | MWcalc RelA (kDa) | MW_exp_ DNA (kDa) | MW_calc_ DNA (kDa) | MW_calc_ Compounds (kDa) |
| --- | --- | --- | --- | --- | --- |
| RelA | 63.5 | 62.3 |  |  |  |
| DNA |  |  | 8.86 | 8.53 |  |
| Epi |  |  |  |  | 0.54 |
| Acla |  |  |  |  | 0.81 |
| RelA + DNA (1:2) | 64.3 |  | 8.86 |  |  |
| RelA + DNA + Epi (1:2:6) | 64.4 |  | 9.23 |  |  |
| RelA + DNA + Acla (1:2:6) | 65.1 |  | 12.5 |  |  |
